# Meta-analysis of the potential of Kazakhstani pork in the global market of meat products: Problems and prospects

**DOI:** 10.14202/vetworld.2022.2705-2714

**Published:** 2022-11-28

**Authors:** Gulmira Karimzhanovna Dambaulova, Svetlana Ivanovna Lilimberg, Vladimir Anatoliyevich Madin, Gainesh Turemuratovna Abdrakhmanova

**Affiliations:** 1 Regional Smart-Center, Non-profit Limited Company A. Baitursynov Kostanay Regional University, Kostanay, Republic of Kazakhstan; 2 Department of Economics, Federal State Budgetary Educational Institution of Higher Education “Chelyabinsk State University,” Kostanay Branch, Kostanay, Republic of Kazakhstan; 3 Department of Software Development and Maintenance, Non-profit limited company A. Baitursynov Kostanay Regional University, Kostanay, Republic of Kazakhstan; 4 Chamber of Legal Consultants of the North Kazakhstan Region, Nur-Sultan, Republic of Kazakhstan

**Keywords:** livestock size, pig farming, pig productivity, pig products, pork export, the profitability of pig farming

## Abstract

**Background and Aim:**

Pig farming is integral in developing Kazakhstan’s animal husbandry as it has shown rapid growth in profit and a high turnover. Industrial pig products have significant global demand, particularly in China and Russia. However, as pig farming is a small-scale industry with insufficient mechanization and automation in Kazakhstan, the costs have increased while the quality of Kazakhstani pig products has decreased due to the simultaneous influx of cheap imported pork into the domestic market. This study aimed to analyze the export potential of the swine industry in the Republic of Kazakhstan and assess its impact on the global pork market.

**Materials and Methods:**

A meta-analysis of open sources was conducted for the period 1990–2020, while forecasting was extended to 2025. Statistical methods for the analysis (construction of time series with equal intervals, calculation, interpretation of average values, and growth rates) were used. Pearson correlation analysis was performed to study the dependence of the productivity of pigs on the category of the pig farming enterprise to establish the relationship between the average live weight of one head of pigs and the share of pigs in agricultural enterprises in the total number of pigs. The polynomial smoothing method was used, and a trend line was built, forecasting the number of pigs in Kazakhstan, the production and sale of pig products, along with their cost and profitability.

**Results:**

This study briefly characterizes the developing situation of the global pork market. We identified and evaluated the development trends in Kazakh pig farming in terms of their impact on the industry’s export potential. Specifically, we determined the following trends: An increase in the actual and projected number of pigs; a decrease in the natural loss of pigs during 2014–2020; the growth of existing and forecast indicators of pork sales in the domestic market; a steady decrease in the share of pigs in small households and an increase in the share of pigs in peasant farms and agricultural enterprises; an increase in the dynamics of the productivity of the pig population; and the growth of actual and predicted profitability of production and sale of pig products. We discussed the main problems that hinder the entry of Kazakhstani pork into the global market: Small-scale farming, insufficient mechanization and automation, and inadequate financial support from the state and banking structures. First, small-scale farming (deduced from the number of pigs by the farm categories) has hampered the small farms’ independent manufacture and use of feed grain (i.e., barley, which is abundant in Kazakhstan), which might reduce costs. Moreover, small-scale farms struggle to comply with the waste disposal rules for pig production and environmental safety norms, which limit the export potential of Kazakhstani pork. Second, insufficient mechanization and automation in feeding and breeding (i.e., use of resource-saving technologies) result in inadequate productivity, decreased competitiveness, and failure to meet global standards. Third, insufficient financial support in the form of grants allocated to purposes other than selection and breeding limits the overall development of Kazakhstani pig farming.

**Conclusion:**

The positive dynamics of the main development indicators of Kazakhstani pig farming efficiently assess the industry’s export potential, according to the country’s leading academic specialists and practitioners in the agricultural sector, especially in light of the difficult epizootic situation. This provides abundant opportunities for export supplies in the global meat market. These indicators include the number of pigs, its natural decline, and structure by farm categories. They also include the production, sale, and profitability of production of pig products and the productivity of pigs. As China and Russia currently have a massive shortage of this product in the domestic markets, they are potential importers of Kazakh pork.

## Introduction

The international market for pig products has several problems, primarily due to a recent African swine fever (ASF) outbreak that hit Chinese pig enterprises in 2018 and rapidly spread across the swine population in the EU countries in 2019 [1–3]. This has affected the dynamics of the main pork exporters globally, causing constant fluctuations in the prices of pig products (mostly downward, hampering the financial condition of the producers) [4]. In 2021, the epizootic rapidly reduced livestock in several Asian and European countries as the disease continued to spread. Although this also threatened the Kazakh pig farming industry, it provided opportunities to develop export for Kazakhstani pork. Today, the Kazakh pig farming industry is not thriving. If there were 3223.8 thousand pigs in 1990, this figure decreased to 816.7 thousand in 2020 by 75% or 3.95 times. Indicators such as productivity, breed, and the number of produced and sold products from pigs indicate a steady decline in the efficiency of the livestock industry 30 years after the post-Soviet development of Kazakhstani livestock.

We highlight various aspects of the functioning of the global pork market and its impact on global food security. Many researchers, considering the theory and practice of the production of pig products, believe that maximum digitalization and automation are required to increase efficiency. Due to the urgent threat of ASF and its impact on the global swine industry [1, 2, 5], researchers are increasingly interested in analyzing the state, trends, and prospects for developing the Chinese swine industry [6–8]. Many Russian and Kazakh scientists have focused on producing and exporting pig products [9–11]. However, the issues related to the potential export of Kazakh pork into the global meat market have not been sufficiently investigated.

Therefore, this study aimed to analyze the export potential of the swine industry in the Republic of Kazakhstan and assess its impact on the global pork market. We also developed measures to increase the export of pig products and the competitiveness of Kazakhstani producers worldwide.

## Materials and Methods

### Ethical approval

This is a meta-analysis study, so, ethical approval is not required for this study.

### Study period and location

The study was conducted from February 2021 to March 2022. The study was conducted at the non-profit, joint stock company “Kostanay Regional University, named after A. Baitursynov” (Kazakhstan).

### Study design

This study followed PRISMA guidelines for meta-analysis study [12]. During the initial stages of the study, we analyzed the situation in the global pork market, identified the current main development trends, and recorded the leading exporters and importers of pork in the world based on secondary data. The next stage of the study focused on analyzing the dynamics and forecasting the development indicators for pig farming in Kazakhstan using quantitative methods.

### Inclusion/exclusion criteria

This meta-analysis was based on desk research. We chose 1990–2020 as the search period, while our forecasting extended to 2025. All the data used in this study were collected from open sources, which were then recorded in a database. The study indicators included the number of pigs and their natural decline, the production and sale of pig products, the structure of the number of pigs by categories of farms, the productivity of pigs, and the profitability of the production of pig products.

### Data extraction

The research information was obtained from the Bureau of National Statistics of the Agency for Strategic Planning and Reforms of the Republic of Kazakhstan and reports by the scientific institutions and organizations on the intensive development of agro-industrial production, namely, LLP “North Kazakhstan Research Institute of Animal Husbandry and Crop Production” (Petropavlovsk), RSE “RPC Animal Husbandry and Veterinary Medicine” (Almaty), the Republican Chamber of Pig farming for all breeds of pigs (the Karaganda region), and the Association “Union of Pig farming Farms of Kazakhstan” (Nur-Sultan).

Additional information was obtained from internet resources, including the information portals of the Federal Center for the Development of Exports of Agricultural Products of the Ministry of Agriculture of Russia “Agroexsport,” “GoFerma.ru” (a website about rearing livestock and vegetables), “News. myseldon.com” (a unified platform that aggregates internet resources from the state bodies of the Ministry of Agriculture of the Republic of Kazakhstan GOV.kz), Main agro news of Kazakhstan (https://world-nan.kz/), Agrobusiness. Kazakhstan of the Republican monthly information, the practical business magazine (https://agbz.kz/o-nas/), and the Kapital.kz Business Information Center.

The analytical sources used for collecting information were as follows: The report on the marketing research for agriculture in Kazakhstan by the Elim Institute for Marketing and Sociological Research dated April 15, 2021; an overview of the pork market of the Eurasian Economic Union member states from 2013 to 2017, compiled by the Department of Agro-Industrial Policy of the Eurasian Economic Commission; an overview of the meat market for each month in 2020 – a joint analytical product of the Center for Industry Expertise of Rosselkhozbank JSC and the Federal State Budgetary Institution “Center for Agroanalytics” dated July 4, 2021; an overview of the meat market for 9 months in 2021 – a joint analytical product of the Center for Industry Expertise of Rosselkhozbank JSC and the Federal State Budgetary Institution “Center for Agroanalytics” dated January 17, 2022; and the analytical review entitled “The meat market in a pandemic: Scenarios for the development of the Russian meat market in 2020–2021” prepared by the National Credit Ratings Agency of the Russian Federation on May 21, 2021.

We also included several legislative acts and government decrees of the Republic of Kazakhstan such as: The Order of the Minister of Agriculture of the Republic of Kazakhstan dated March 15, 2019, No. 108 and registered with the Ministry of Justice of the Republic of Kazakhstan on March 20, 2019, No. 18404 “On the approval of the rules for subsidizing the development of livestock breeding, increasing the productivity, and quality of livestock products;” the Law of the Republic of Kazakhstan dated July 9, 1998, No. 278-I “on livestock breeding” (with amendments and additions as of January 7, 2021); the decree of the Government of the Republic of Kazakhstan dated December 28, 2020, No. 898 on the amendments and additions to the Decree of the Government of the Republic of Kazakhstan dated July 12, 2018, No. 423 “on the approval of the state program for the development of the agro-industrial complex of the Republic of Kazakhstan for 2017–2021;” the concept of development of the agro-industrial complex of the Republic of Kazakhstan for 2021–2030; the state program for the development of the agro-industrial complex of the Republic of Kazakhstan for 2017–2021; and the national project for the development of the agro-industrial complex of the Republic of Kazakhstan for 2021–2025.

### Statistical analysis

We used statistical methods for the analysis (construction of time series with equal intervals, calculation, interpretation of average values, and growth rates). We applied a Pearson correlation analysis using SPSS statistics version 26.0 (IBM Corp., Armonk, NY, USA) to study the dependence of the productivity of pigs on the category of the pig farming enterprise to establish the relationship between the average live weight of one head of pigs and the share of pigs in agricultural enterprises in the total number of pigs.

We used the polynomial smoothing method and built a trend line, forecasting the number of pigs in Kazakhstan, the production and sale of pig products, along with their cost and profitability. Our results indicate a positive trend in the study indicators for the prospective period. The results were processed using the abovementioned statistical methods and presented using graphs and tables. Microsoft Excel was used for analyzing, forecasting, and plotting graphs.

## Results

**Figure-1:**
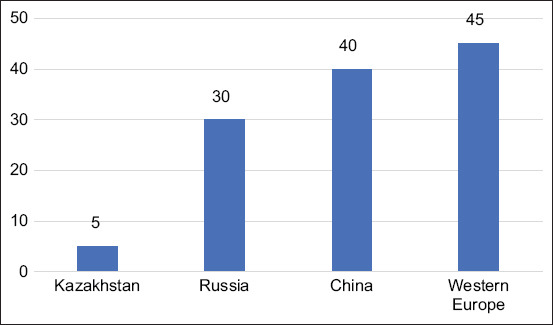
Pork consumption in the world in 2020, kg per capita.

For any kind of product, the volume of export potential is invariably linked to how much it meets the domestic market needs. Since Kazakhstan is a Muslim country, it traditionally has a low demand for pork as a food product. Therefore, pork is not the most popular type of meat in Kazakhstan, especially compared with other non-Muslim states [13, 14] (Figure-1).

**Figure-2:**
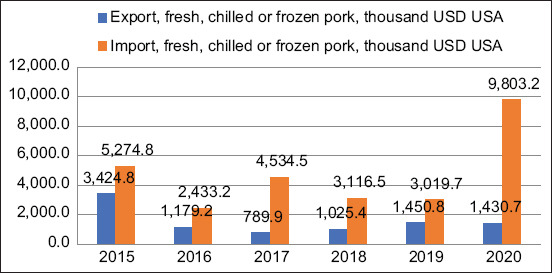
Dynamics of exports and imports of pork in Kazakhstan.

However, the multi-nationality of the Kazakhstani population determines the presence of a diverse demand for all meat products, including pig products. Therefore, pork is still in demand in Kazakhstan, where approximately 20% are non-Muslims. Unfortunately, currently, Kazakhstani pig breeders have nothing to export. Even the needs of the domestic market are fulfilled mainly by the import of pig products (Figure-2).

**Figure-3:**
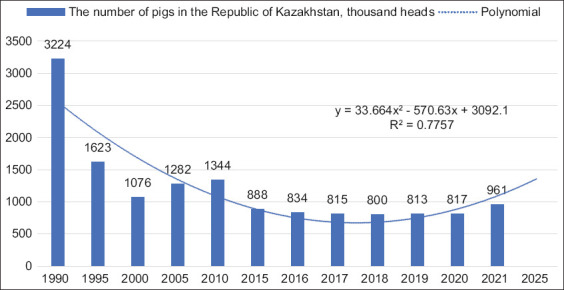
The dynamics and forecasting of the number of pigs in the Republic of Kazakhstan using a polynomial trend model.

In 2020, with exported products worth 1430.7 thousand US dollars, Kazakhstan had to import pork for a total of 9803.2 thousand US dollars (almost seven times the volume of exports) to cover the domestic market needs. When the Kazakhstani market economy was formed in 1990–2000, Muslims were predominant among the Kazakhstani population. As a result, the demand shifted toward horse meat and lamb, which are traditional in Kazakhstan. Due to this, the government ignored the problems of pig farming, resulting in the current crisis in the Kazakh pig industry. However, historically speaking, pig farming in the Kazakh state was rapidly developed in the Kazakh SSR, as evidenced by the indicators of the dynamics of the number of pigs, which were more than 3 million heads in 1990 (Figure-3).

**Figure-4:**
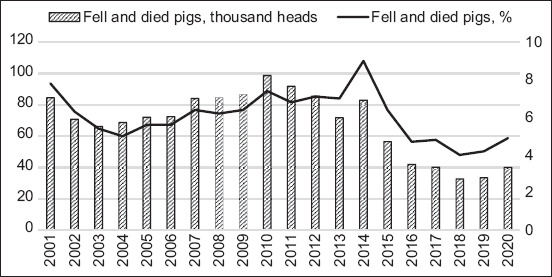
The dynamics of indicators of natural decline in the number of pigs in the Republic of Kazakhstan.

The number of pigs in Kazakhstan decreased significantly from 1990 to 2020. For the analyzed period of 1990–2020, the number of animals decreased significantly in 1995 (50% compared to 1990). In 2005 and 2010, this figure increased by 19% and 25%, compared to 2000. Since 2011, livestock has steadily declined again, reaching its lowest level of 800 thousand heads in 2018. Starting from 2019, it has shown an upward trend as it increased by 20% in 2021 compared to 2018. The analysis forecasts the number of pigs up to 2025. The construction of a polynomial trend line indicates the upcoming increase in the number of pigs in Kazakhstan with a degree of approximation reliability equal to 0.772 and the second degree of the polynomial, close enough to actual values. According to the forecast, the number of livestock in 2025 might approach 1500 thousand heads, which positively characterizes the export potential of Kazakhstani pig farming. The state of development of the livestock industry, its increase or decrease, is also characterized by the indicators of animals’ natural loss (case) (Figure-4).

**Figure-5:**
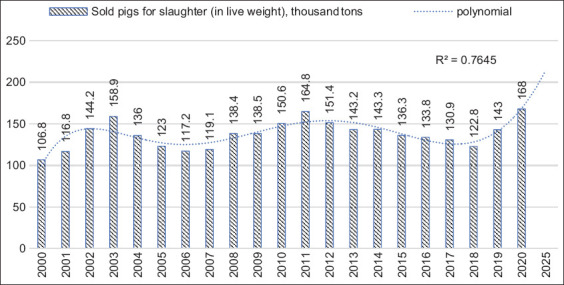
The dynamics and forecasting of production and sales of pig products in the Republic of Kazakhstan using a polynomial trend model.

An analysis of the dynamics of this indicator in the pig industry in the Republic of Kazakhstan also positively assessed the industry’s export potential since a decrease in natural losses suggests a promising increase in the livestock number. The highest rate was observed in 2001 (7.8%) and 2010 (7.3%) due to the industry’s low level of zootechnical service, irrationally organized animal feeding, and inefficient technical and technological system to manufacture pig products. The dynamics of pork sold for slaughter in live weight shows an upward trend in 2001–2020, although insignificant (Figure-5).

**Figure-6:**
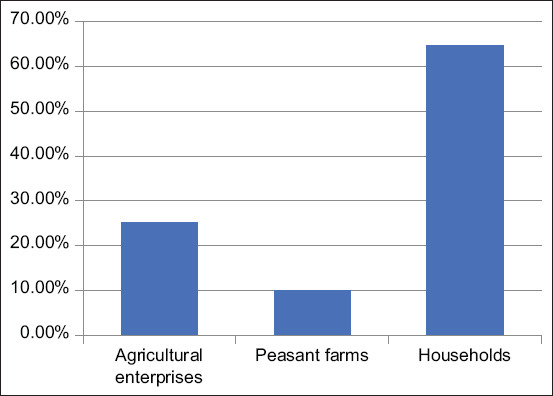
The structure of the number of pigs by categories of farms of the Republic of Kazakhstan in 2020.

The peak of production and sales of pig products occurred in 2004 (158.9 thousand tons) and 2012 (164.8 thousand tons). In 2020 (the last year of the analyzed period), this figure was 168 thousand tons, exceeding the previous years’ figures. The average annual growth rate of this indicator was 0.788%. Consequently, pork production in Kazakhstan shows an upward trend, which was also confirmed by the prediction results for the future production of pig products using a polynomial trend model. According to the forecasting results (with the accuracy of the approximation of 0.7645 and the 6^th^ degree of the polynomial), the production of pig products might exceed 220 thousand tons in 2025. However, due to the insufficiently high level of reliability, it is possible to doubt the results of applying this statistical forecasting method and to assume the possibility of achieving a higher value of this indicator – up to 300 thousand meat per year, which will not only cover the domestic needs for pork but also increase its export. Studies show that small farms are mainly engaged in pig farming in Kazakhstan, which is typical for all livestock industries (Figure-6).

Small commodity producers cannot afford to maintain proper zootechnical records, breeding and selection work, and provide an appropriate forage base, which hinders the effective development of the industry [15]. However, changes in trend were observed when the dynamics of the pig farming process were analyzed for a longer period (Table-1).

**Table-1:** Structural changes in the number of pigs by categories of farms of the Republic of Kazakhstan, %.

Farm categories	Year 2001	Year 2006	Year 2011	Year 2016	Year 2020
In all categories of farms	100	100	100	100	100
In agricultural enterprises	9.6	13.0	17.5	30.8	29.6
In peasant farms	2.9	5.4	7.1	11.9	9.8
In households	87.5	81.6	75.4	57.3	60.6

**Figure-7:**
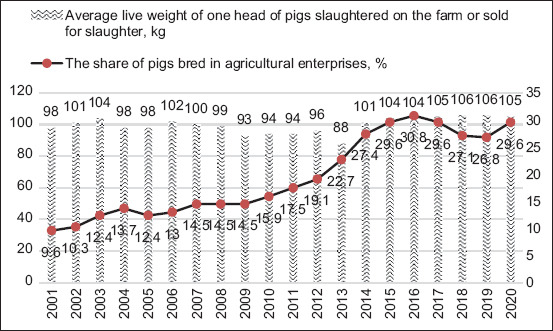
Analysis of the dynamics of pig productivity in the Republic of Kazakhstan and the share of pigs bred in agricultural enterprises.

The data on the structural dynamics of the number of pigs in Kazakhstan show a steady decrease in the share of pigs in small households and an increase in the share of pigs in peasant farms (6.9% points in 2020 compared to 2001) and agricultural enterprises (by 20% points in the same period). Therefore, in the future, we can assume that the number of large- and medium-sized pig breeding enterprises will increase in the Republic, which will increase the level of automation, mechanization, and digitalization of the industry, which will ultimately favor exports. The analysis of pig productivity confirms the earlier conclusion about the benefits of breeding pigs in large- and medium-sized farms (Figure-7).

The productivity of pigs in households is 15%–20% lower than the animals bred in agricultural enterprises. During the study, a correlation analysis of the interdependence between the indicators presented in Figure-7 was performed: The average live weight of one head of pigs and the share of pigs of agricultural enterprises in the total number of pigs. The calculated correlation coefficient was 0.446079, significant at p < 0.05. In other words, 45% of the productivity of pigs depends on where the pigs are kept and bred. Consequently, an increase in the export of pig products is possible only with the expansion of agricultural pig breeding enterprises and the development of industrial pig breeding. This also fully meets the requirements for pork import by China.

**Figure-8:**
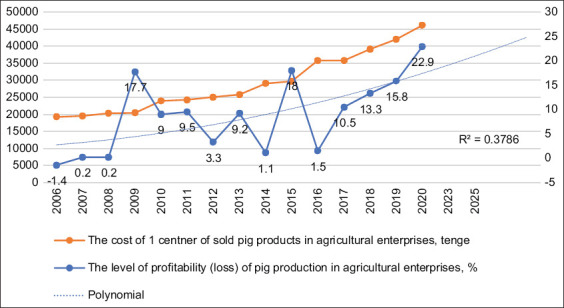
Analysis and forecasting of the profitability of the production of pig products in the Republic of Kazakhstan using a polynomial trend model.

The most important economic indicator characterizing the efficiency of the industry development is the cost of production and profit from its sale. The effectiveness of production is manifested in the profitability indicator. Figure-8 explains the dynamics of the profitability of the production of pig products and forecasts its future – the cost of one center of production of pig products in the analyzed period. The average cost growth rate for 2006–2020 was 1.129. Simultaneously, the level of profitability of pig production in the same period changed in different directions. However, the polynomial smoothing of this indicator during the analyzed period shows an increasing trend in the profitability of pig farming, despite a similar change in the cost.

## Discussion

### Problems of modern pig breeding

Recently, the circumstances in the global pork market have changed significantly as pig farming has been affected by the epizootic. Animal husbandry is a high-risk industry. Therefore, the evolution and dynamics of meat food market development depend on factors such as the prevalence of animal diseases, sanitation and hygiene, and the introduction of new environmental standards [16]. Numerous outbreaks of ASF adversely affected pork production in several countries between 2018 and 2020 [1–3], especially in China, the world’s largest pork producer [6]. The Chinese pig population in 2019 decreased by approximately 180 million, and according to Zhang and Wang, it will take much time to restore it [17]. In 2020–2021, the US, Canada, and Brazil became the world’s top pork importers, while China turned from an exporter to a significant pork buyer in the global market.

The unstable pricing policy is a characteristic feature of the modern global pork market. China, the largest pork buyer, started restricting pork imports from several European countries due to the spread of the African plague in these countries. Thus, in 2021, the persistent epizootic situation in this region and the following restrictions on exports resulted in an excess supply in the market [18]. Naturally, this decreased pork prices in 2021 by an average of 15% compared to 2020. However, according to global forecasts and those made by Chinese scientists, the demand and prices of pork will increase between 2020 and 2030 [4, 8, 19]. The Chinese market continues to experience a massive shortage of pork. However, Kazakhstan, which borders China, is one of the few countries that are officially recognized to be free from ASF by the World Organization for Animal Health (formerly the Office International des Epizooties). Hence, Kazakhstan can freely export pork to China. Sanctions against Russian-made products introduced in 2022 also gave Kazakhstan access to the European market for pig products.

Pig breeding in Kazakhstan currently has several challenges, mainly due to the lack of established fodder production, poor breeding techniques, inadequate livestock optimization, and an insufficiently developed legislative framework [13, 20, 21]. Some authors believe that there is practically no civilized market for selling pig products in Kazakhstan. Moreover, the work aimed at expanding the domestic and export trade market for pig products is poorly executed [15]. However, Fedorov *et al*. [22] reported that industrial pig breeding is currently one of the most high-tech sectors of the agro-industrial complex.

Most Kazakhstani and international authors have emphasized that the volume of livestock products can be enhanced using innovative technologies, digitalizing the farms, and developing and scientifically validating rational options and technical solutions for inexpensive, environmentally friendly meat production [7, 9, 23]. The authors state that the transfer and adaptation of highly efficient technologies based on automation and digitalization of production processes in animal husbandry for meat production are required to meet the existing high demand for Kazakhstani meat products in the domestic and foreign markets, considering the objective conditions in different regions of the country.

Yesengaliyeva *et al*. [15], reported that inadequate labor productivity due to substandard automation and the digitalization of technological processes seriously affects modern pig breeding. Several studies have emphasized the need to develop a scientific justification and master modern technologies for inexpensive, environmentally friendly, and competitive production in the pig industry [24, 25]. Mikhailova believes that using resource-saving technologies are critical for profitability in the pig industry [26].

Most studies on pig farming state that a comprehensive, technical, technological, and organizational improvement in production using the latest scientific achievements and best practices is necessary to increase the efficiency, export potential, and competitiveness of pig products globally.

### Main trends in the development of the pig farming industry

Based on our analysis, we suggest trends in the development of the industry and positively assess the global export potential of Kazakhstani pig farming. First, this study forecasts a potential increase in the actual and projected number of pigs. This can be attributed, to some extent, to the possible growth of genetically healthy, highly productive, and locally adapted livestock of pigs due to the restoration of the Kazakhstani genotype. The growth of the number of pigs would support the claimed potential of the export of Kazakhstani pork, enabled by the worldwide losses due to the ASF outbreak [27].

Second, we forecast a decrease in the natural loss of pigs in the dynamics of 2014–2020. The main reasons for the natural loss of pigs include irrationally organized animal feeding and inefficient technical and technological system for producing pig products. This prediction seems valid since there have been improvements in technological support [28].

Third, the growth of actual and forecasted indicators such as production volume, sales volume, and the average annual growth rate in the domestic market indicates the possibility of a short-term increase in the volume of produced pig products. However, this trend might be considered highly credible due to the nature of pig production and sales and their dependence on various external and internal factors.

Fourth, a steady decrease in the share of pigs in small households and an increase in the share of pigs in peasant farms and agricultural enterprises might contribute to the active development of the cooperative sector in the Kazakhstani livestock sector and the increased use of the “anchor partnership” model. This provides a solid organizational and economic base for pig farming. Besides improving productivity, these “anchor partnerships” would both financially and non-financially benefit from the WTO “Green Box” support [29].

Fifth, an increase in the dynamics of the productivity of the pig population was found to be correlated with the enlargement of pig farms. Since peasant farms and enterprises are expected to grow, pig production’s productivity is also anticipated. For instance, the pig farms in the Almaty region have benefited from the outcomes of the scientific program of the Kazakh research institute of livestock and fodder production. They are planning to export their production to China [30].

Finally, the growth of actual and predicted profitability of production and sale of pig products could be reinforced by a steady supply of independently produced fodder thanks to the developed grain production in the country and the possibility of meat conversion, such as the export of meat products instead of feed grain. The use of Kazakh fodder reduces the farmers’ dependency on currency volatility (USD, EUR, RUB), at least from the perspective of feed [31, 32].

### Problems and measures to increase the export of Kazakhstani pig products

We believe that pig farming can potentially play a leading role in developing Kazakhstan’s animal husbandry, one of the fastest-growing industries. However, it is necessary to consider the factors that negatively impact the industry’s development. As shown in this study, the main negative factor is the production of the predominant part of the products in households, small-scale pig farming, with an increase in the productivity of pigs in large enterprises. Low, insufficiently developed cooperation and integration of Kazakhstani pig breeders hamper the quality of pig products, seriously affecting its export in the global meat market. In addition, due to small-scale farming, technical and technological problems arise, such as a low level of mechanization of the feed base, non-compliance with the technology of feeding pigs, the use of outdated breeding techniques based on minimal costs, and lack of proper monitoring of performance indicators of pig farms. The development of the industry is not favored by the lack of financial support for pig farms from the government and banking structures, which is a consequence of unhealthy populism and criticism of pig farming by some public figures. At present, the financial support of the industry from the state is directed only to the purchase of breeding animals abroad and (or) executing selection and breeding work. Despite the insignificant amount of funds allocated by the state, selection and breeding activities in Kazakhstani pig farming are extremely important and needed, especially in the absence of a breeding and hybrid center in the country [14]. During the Soviet period, highly producing genotypes such as the Semirechenskaya breed and the Aksai black-and-white group were bred in Kazakhstan due to favorable local climatic and forage conditions. Unfortunately, these breeds are now almost extinct.

The abovementioned reasons have caused a multidirectional change in the profitability of the production of pig products with a steady increase in cost. However, the conditions of Kazakhstani agricultural output favor a reduction in the cost of meat [33]. Barley is abundantly grown in Kazakhstan annually and is exported abroad as forage. This barley can be used independently by Kazakhstani pig producers as compound feed – the main type of feed for pigs [34]. This enables the so-called meat conversion – to export not the barley itself but the pig products produced with it, which can significantly increase the country’s export earnings. In addition, using feed grain in their production will allow Kazakhstani pig breeders to reduce the cost of pork, making its price attractive in the global market.

Organizing feed production directly on the farm is ideal, which can only be afforded by large- or medium-sized pig breeding enterprises, while small farms are forced to purchase mixed fodder of questionable quality from the outside. The problems of small-scale pig farming in Kazakhstan can be solved by developing a cooperative sector, particularly by creating associations of small farms based on the so-called “anchor partnership,” which cumulatively make a large meat processing enterprise. This mutually beneficial cooperation will provide new opportunities for all its participants by reducing costs and receiving government subsidies and, therefore, enable not only an increase in the number of pigs but also strengthen the feed base, which will positively impact the quality and competitiveness of pig products [10].

**Figure-9:**
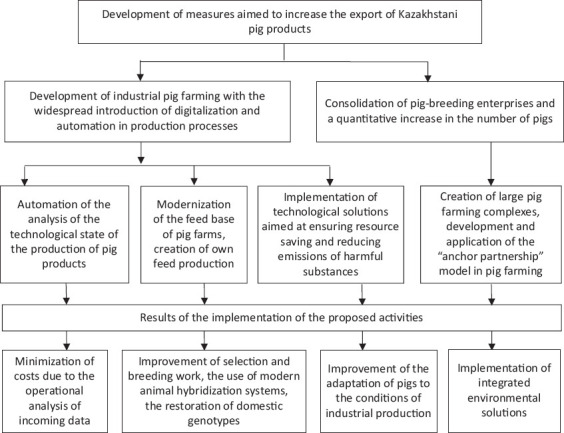
Development of measures to increase the export of Kazakhstani pig products.

The task of maintaining the profitability of the industry is closely related to environmental safety. The problems of pig farming today are not only due to organizational or technical and technological reasons but are also associated with the environmental pollution caused by the waste products of pigs [35]. This, in turn, is another factor in favor of the expansion and cooperation of pig farms since only large pig farms can have treatment facilities. In addition, compliance with the rules for waste disposal from pig production is an important factor in the growth of export potential. The violation of these rules can cause the spread of infection and damage the environment [23]. This problem requires new technological solutions to ensure resource-saving emissions of harmful substances. Figure-9 shows the measures developed by the authors to increase the export of Kazakhstani pig products.

The informatization process is quite active in all branches of animal husbandry. Any substantial pig farming complex should have touch sensors and digital video cameras in its facilities. However, this process should become more global. Industrial pig farming should incorporate automated production management systems based on artificial intelligence [36, 37]. Moreover, the negative impact of the digitalization of pig production on the conditions of keeping pigs should be avoided.

## Conclusion

Kazakhstani industrial pig farming has a significant export potential. The dynamic growth in the number of pigs, their productivity, reduction in natural loss, and increase in profitability, and an optimistic forecast for the growth of these indicators in the prospective period indicate that Kazakhstani pig breeder can potentially export their products in the global meat market. The attractiveness of Kazakhstani pork in the global meat market is determined by favorable prices, which might be lower than international pork prices.

The epizootic, which has developed in the global pork market and resulted in bans on pork exports from ASF-affected countries, is the main driver of the growth of the export potential of Kazakhstani pig farming. In Kazakhstan, no cases of ASF infection have been registered in animals. At the end of 2020, the Committee for Veterinary Control and Surveillance of the Republic of Kazakhstan developed a plan of organizational, veterinary, sanitary, and economic measures to prevent disease occurrence. Notably, it introduced restrictions on the import and transit of pig farming and related products from the regions of the Russian Federation through Kazakhstan.

Moreover, Kazakhstan is currently gaining opportunities to enter the global market with its products, especially for exporting pork to China and Russia. Previous studies have shown that the export potential of Kazakhstani pig farming can be 250–300 thousand tons of pork per year, which will bring the state a billion in revenue. Thus, Kazakhstani pork producers, with appropriate state support, can optimistically anticipate and not only bring high profits to the economy of Kazakhstan but also contribute to maintaining global food security.

In summary, the priorities for developing the swine industry in Kazakhstan should include measures to increase the export of Kazakhstani pig products, enable the use of practical tools for digitalization and production automation, implement solutions for the modernization of the forage base, develop technological projects aimed at ensuring resource-saving, reduce emissions of harmful waste from the industry into the environment, and methodically construct the organizational and economic model of “anchor partnerships.”

## Authors’ Contributions

GKD: Study conception and design and critical revision of the manuscript. SIL: Analysis and interpretation of data and drafted the manuscript. VAM: Acquisition of data, analysis, and interpretation. GTA: Acquisition of data and drafted the manuscript. All authors reviewed the results and approved the final version of the manuscript.
